# In-Depth Profiling of the Peripheral Blood Mononuclear Cells Proteome for Clinical Blood Proteomics

**DOI:** 10.1155/2014/129259

**Published:** 2014-03-03

**Authors:** Saša Končarević, Christopher Lößner, Karsten Kuhn, Thorsten Prinz, Ian Pike, Hans-Dieter Zucht

**Affiliations:** ^1^Proteome Sciences R&D GmbH & Co. KG, Altenhöferallee 3, 60438 Frankfurt am Main, Germany; ^2^Proteome Sciences Plc, Coveham House, Downside Bridge Road, Cobham KT11 3E, UK

## Abstract

Peripheral blood mononuclear cells (PBMCs) are an easy accessible cellular part of the blood organ and, along with platelets, represent the only site of active gene expression in blood. These cells undergo immunophenotypic changes in various diseases and represent a peripheral source of monitoring gene expression and posttranslational modifications relevant to many diseases. Little is known about the source of many blood proteins and we hypothesise that release from PBMCs through active and passive mechanisms may account for a substantial part of the plasma proteome. The use of state-of-the-art proteomic profiling methods in PBMCs will enable minimally invasive monitoring of disease progression or response to treatment and discovery of biomarkers. To achieve this goal, detailed mapping of the PBMC proteome using a sensitive, robust, and quantitative methodological setup is required. We have applied an indepth gel-free proteomics approach using tandem mass tags (TMT), unfractionated and SCX fractionated PBMC samples, and LC-MS/MS with various modulations. This study represents a benchmark in deciphering the PBMC proteome as we provide a deep insight by identifying 4129 proteins and 25503 peptides. The identified proteome defines the scope that enables PBMCs to be characterised as cellular major biomarker pool within the blood organ.

## 1. Introduction

Peripheral blood mononuclear cells (PBMCs) constitute the cellular part of the blood organ containing all blood cells with a round nucleus. PBMCs are mainly comprised of monocytes, T cells, B cells, natural killer (NK) cells, and dendritic cells. Thus, the PBMCs contain different cell types that play important roles in the immune system monitoring immune-relevant events and respond in an inflammatory manner [1]. In recent years PBMCs have received growing attention as surrogate markers of several diseases. For example, in vitro data describe the response in PBMCs upon contact with diseased cells [2]. PBMCs can be obtained relatively easy from routinely collected blood samples, and therefore they provide direct access to physiologically relevant (immune) proteins without the well-known analytical difficulties of native human plasma originating from the presence of highly abundant proteins [3]. So far, most Omics studies utilising PBMCs were transcriptional profiling experiments in the context of inflammatory (e.g., preeclampsia, rheumatoid arthritis, and chronic pancreatitis) and malignant (e.g., chronic lymphocytic leukaemia and renal cell carcinoma) diseases [4–8]. Although these studies revealed a number of differentially regulated genes at the mRNA level, such observations cannot often directly be translated into protein abundance [9]. Furthermore, posttranslational modifications and processing of proteins play a significant role in regulating cellular response pathways to disease and can greatly influence the functions and activities of proteins in a given sample. This protein-specific information can play an essential role in defining new targets and biomarkers for a disease but is often lost when proteins are released into plasma. Prior to the present study, proteomic investigations using PBMCs have revealed only a limited set of proteins. In 2008, Vergara et al. established a 2D database including 174 proteins [10]. Haudek-Prinz et al. have published a proteomics study where they comprehensively characterize a proteome signature of inflammatory-activated PBMCs. They used two methodological approaches with 2D-PAGE and MS-shotgun analysis and described the detection of 1774 PBMC proteins [1, 11]. Wang and coworkers analysed PBMCs of systemic lupus erythematosus patients with isobaric tagging for relative and absolute protein quantification (iTRAQ) and found 67 out of 452 totally identified proteins to be altered in their expression [12]. Recently, Maccarrone et al. have published the identification of the PBMC proteome with 514 proteins with at least two nonredundant peptides [13].

In order to broaden the scope for a proteomics biomarker discovery, there is need for technical and methodical improvement to achieve a more detailed picture of the proteomic patterns in PBMCs. This will greatly enhance the prospect of discovering valid biomarkers in PBMCs. This particularly is true when considering PBMC-derived proteins that are naturally present only at very low concentrations in serum or plasma and may represent important biomarkers.

To achieve the goal of providing an in-depth proteome coverage of human PBMCs, we have conducted a study that combines the LC-MS analysis of unfractionated as well as SCX fractionated human PBMC samples. Further we have varied and optimized RP-LC gradients and length, applied time and mass dependent exclusion of previously identified peptides, run several analytical replicates, and used gas phase fractionation. In order to instantaneously provide a means that enables efficient and accurate quantification of the peptides and proteins identified in this in-depth analysis of the PBMC proteome, the workflow is based on the use of isobaric labelling reagents, that is, tandem mass tags (TMTs) [14]. Finally, we have characterized the PBMC proteome by utilizing gene ontology as well as pathway annotations and also compared it to the plasma proteome (HPPP [15]) in order to illustrate that the two different sample types (that can be obtained from blood as the same source sample) harbor a different proteomic information basis. This highlights the benefits of using PBMCs for clinical proteomics to enable monitoring of disease progression or response to treatment and discovering of valid biomarkers in this blood cellular compartment.

## 2. Materials and Methods

### 2.1. Preparation of PBMC Lysates

We have used commercially available PBMC cells as samples (Astarte Biologics, LLC, Redmond, USA, Catalogue number 1000, Lot number 740MA11). These are collected from healthy donors by leukapheresis and are further purified on Ficoll-sodium metrizoate density gradients. The cells are washed extensively and frozen in a solution of 10% DMSO and 2% human serum albumin in phosphate buffered saline.

Typically they are composed of 50–70% T cells, 5–15% B cells, 10–20% monocytes, 2–10% natural killer cells, and the remainder of platelets. The PBMC sample used for analysis is from a healthy 56-year-old Caucasian male (vendor information). After thawing PBMCs were centrifuged and washed with PBS. The resulting cell pellets were subjected to ultrasonic lysis in ice-cold TEAB buffer containing TCEP and SDS [16].

### 2.2. TMT Workflow

For the application of the TMT technology, 300 *μ*g protein extract was diluted to a protein concentration of 1 *μ*g/*μ*L by adding 100 mM TEAB buffer containing 0.1% SDS. Proteins were reduced for 60 min at 55°C by the addition of 16 *μ*L of 20 mM aqueous TCEP and subsequently alkylated for 60 min at room temperature by addition of 16.5 *μ*L 150 mM iodoacetamide in acetonitrile. To digest proteins, 30 *μ*L of 0.4 *μ*g/*μ*L trypsin (sequencing grade modified trypsin, Promega) in 100 mM TEAB was added and the sample was incubated at 37°C for 18 h. Subsequently, digested proteins were labelled by adding 121 *μ*L of a 60 mM TMTzero reagent (available through Thermo Scientific) solution in acetonitrile for 1 h at room temperature. To reverse occasional labelling of Tyr, Ser, and Thr residues, 25.5 *μ*L of an aqueous hydroxylamine solution (5%, w/v) was added and incubated for 15 min at room temperature.

The TMT-labelled sample was diluted to 2 mL with 5% ACN containing 0.1% TFA and desalted with Oasis HLB cartridges (Waters) according to manufacturer's instruction; however, elution of peptides was performed with 750 *μ*L of 50% ACN containing 0.1% TFA.

The eluate was used immediately for either SCX purification or SCX fractionation.

### 2.3. SCX Purification

The eluate from the HLB cartridge was directly loaded onto self-made cartridges (CHROMABOND empty columns of 15 mL, Macherey-Nagel, filled with 650 *μ*L SP Sepharose Fast Flow, Sigma) and, after washing with 4 mL of 25% ACN and 0.1% TFA, the peptides were eluted with 2 mL of 25% ACN and 400 mM ammonium acetate. The samples were dried with a vacuum concentrator.

### 2.4. SCX Fractionation

The eluate from the HLB cartridge was loaded directly onto a Polysulfoethyl A column (4.6 × 100 mm, 5 *μ*m, 200 Å, PolyLC) attached to a Waters 2695 HPLC. The SCX gradient was run with a flow rate of 2 mL/min and was as follows: 0–10 min: 5 mM KH_2_PO_4_ in 25% acetonitrile (pH 3 adjusted with H_3_PO_4_); 10–43 min: linear gradient from 0 to 250 mM KCl in 5 mM KH_2_PO_4_ in 25% acetonitrile (pH 3). One-minute fractions were collected during the gradient (10–43 minutes). Based on the UV absorbance at 214 nm, ten fractions were selected and dried with a vacuum concentrator.

### 2.5. MS Data Acquisition and Analysis

Dried samples were reconstituted using 2% acetonitrile and 0.1% formic acid in 97.9% water. 1 *μ*g of unfractionated sample or 5 *μ*g equivalent of each SCX fraction was measured on a LTQ Orbitrap Velos (Thermo Scientific) coupled to an EASY-nLC II (Proxeon). Samples were trapped on a 0.1 × 20 mm column packed with ReproSil C18, 5 *μ*m (Dr. Maisch). After loading and washing of the samples, the separation was run on a 0.075 × 150 mm self-packed column with ReproSil C18, 3 *μ*m (Dr. Maisch). For the two-hour (4, 6, and 8 hour) method, a 90 minutes (215, 335 or 455 minutes) gradient ranging from 5% of acetonitrile (0, 10, or 15% for the optimized gradients) in 0.1% formic acid/water to 30% of acetonitrile at a flow rate of 300 nL/min has been used, respectively. MS spectra ranging from 350 to 2000 Th (300–1500, 300–536, 531–679, 674–840, and 835–1500 for gas phase fractionation) were acquired in the Orbitrap at a resolution of 30000 (automatic gain control target of 1E6 and maximum ion fill time at 500 ms), and the ten most intense ions with a minimal required signal of 10000 were subjected to MS/MS by HCD fragmentation in the Orbitrap at 7500 resolution (normalized collision energy at 45, isolation width of 2.0 Th, 0.1 ms activation time, automatic gain control target of 5E4, and maximum ion fill time at 500 ms). For exclusion runs, the top 10 most intense ions not on the reject mass list which was based on previous runs have been selected. Unassigned and +1 charged ions were excluded for MS/MS and a dynamic exclusion list with a duration of 30 seconds and 10 ppm exclusion mass width was applied. An overview over conducted LC-MS runs is provided in Table 1.

MS data analysis was conducted using Proteome Discoverer 1.4. Sequest HT was used with the UniProt SwissProt human database (2013_03) allowing full tryptic activity with a maximum of two missed cleavage sites, a precursor mass tolerance of 20 ppm, and a fragment mass tolerance of 0.02 Da. Further, variable modifications of methionine oxidation (+15.995) and static modifications of TMT at any N-terminus and K (+224.152) as well as carbamidomethylation of cysteines (+57.021) were allowed. Percolator and a decoy database were used resulting in an acceptance criterion of below 1% peptide positive false discovery rate (pFDR). Peptide and protein grouping was enabled applying the strict maximum parsimony principle. All protein, peptide identifications, and peptide spectral matches (PSMs) are available in the supplementary data file S1 (available online at http://dx.doi.org/10.1155/2014/129259).

### 2.6. Bioinformatic Analysis

A plasma protein list, consisting of 1929 high confidence proteins, has been downloaded from the Human Plasma Proteome Project (HPPP) data central at PeptideAtlas (http://www.peptideatlas.org/hupo/hppp/, date August 14, 2013) [15]. Protein lists of PBMC and plasma (HPPP) were submitted to the DAVID bioinformatics resources tool (version 6.7, http://david.abcc.ncifcrf.gov/) for protein annotation [17, 18]. Of available information the data originating from the Gene Ontology (GO) database and the Kyoto Encyclopedia of Genes and Genomes (KEGG) pathways were further analysed.

## 3. Results and Discussion

Conventional proteomic analyses of samples of blood origin (e.g., plasma and serum) most often comprise mainly high-abundant proteins and often lack the detection of low-abundant proteins. However, low-abundant proteins from blood can represent valuable biomarker candidates, such as proteins of disease specific pathways (e.g., Huntington's, Parkinson's, and Alzheimer's disease) [19].

Detection of low-abundant proteins can be achieved by a customized sample selection and efficient preparation method paired with an extensive sample fractionation methodology. Finally, the protein detection and quantification has to be highly sensitive by using an appropriate LC-MS/MS setup. This is greatly assisted through the use of isobaric TMT labelling reagents allowing for a multiplex quantitative mass spectrometric analysis of samples, which decreases artificial deviations and increases throughput.

In order to comprehensively characterize PBMC samples, we have applied several preparative as well as analytical variations. The PBMC sample, used in this study, is from a healthy individual; thus, we assume that proteins detected here are generally quantifiable in PBMC samples with our method. On the preparative side we have analysed SCX fractionated samples as well as unfractionated samples and on the analytical side we have included analytical repeats, exclusion list runs (excluding previously identified peptides from MS/MS selection), gas phase fractionation (variation and adaption of m/z windows), extended gradient length (from two to four, six, and eight hours), and optimization of RP gradients for SCX fractions [20, 21]. In Table 1, we have recapitulated all LC-MS acquisitions. Therefore we have depicted the fractionation method, if applied, and the used liquid chromatography gradient, as well as modifications in the mass spectrometric method.

**Figure 1:**
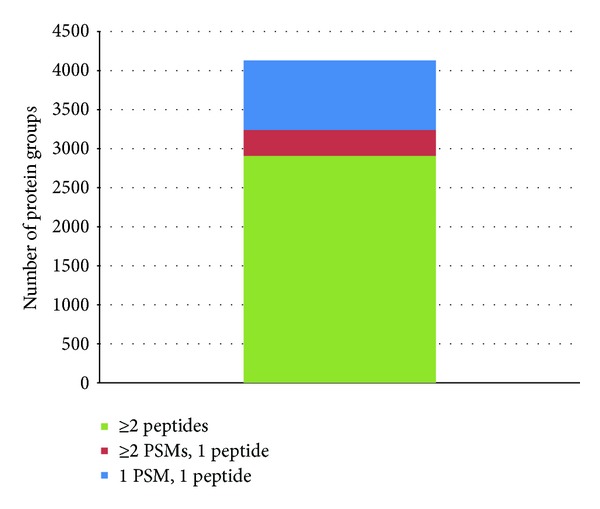
Number of identified protein groups consisting of at least one peptide spectral match (PSM), two PSMs, or two peptides.

By this approach we have identified the total number of 4129 protein groups consisting of 25503 peptide groups and 203665 peptide spectral matches (PSMs) (see Supplemental Table S1). Out of the total number of proteins, 2909 protein groups have been identified with at least two peptides, 331 with at least two peptide spectral matches, and 889 protein groups with at least one peptide spectral match fulfilling a pFDR of lower than 1% (see Figure 1).

**Figure 2:**
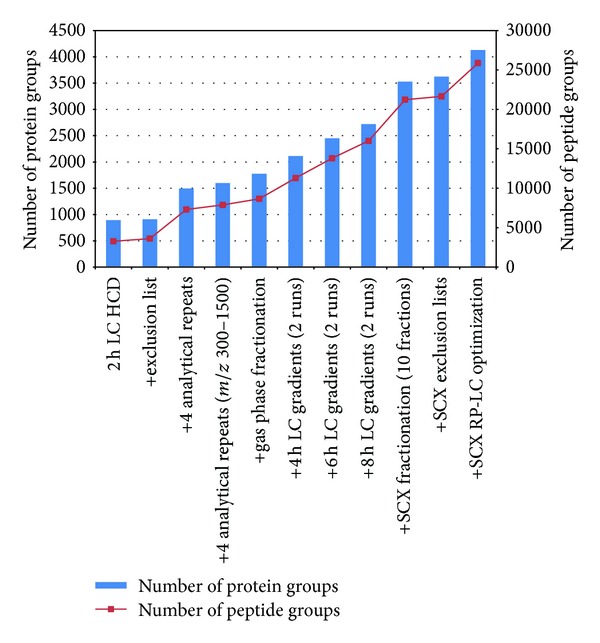
Cumulative protein and peptide group identifications considering various preparative and analytical variations.

By using a whole set of methodological variations we have achieved significant increases of identified proteins. The most significant increase in the number of identifications was resulting from analytical repeat measurements, LC gradient extension, and SCX fractionation, as well as RP-LC optimization for SCX fractions (see Figure 2). Additionally, the resulting repetitive identification of peptides in the different runs increases the number of peptide spectral matches for one peptide and thus improves the overall quality of the results. By this we have extended the catalogued proteome of human PBMCs by more than double the number [11]. Our data set containing all identified proteins and peptides connected to the respective methodical setup can serve as a starting point for the design of future studies, where experiments focusing on selected proteins of interest shall be conducted.

**Figure 3:**
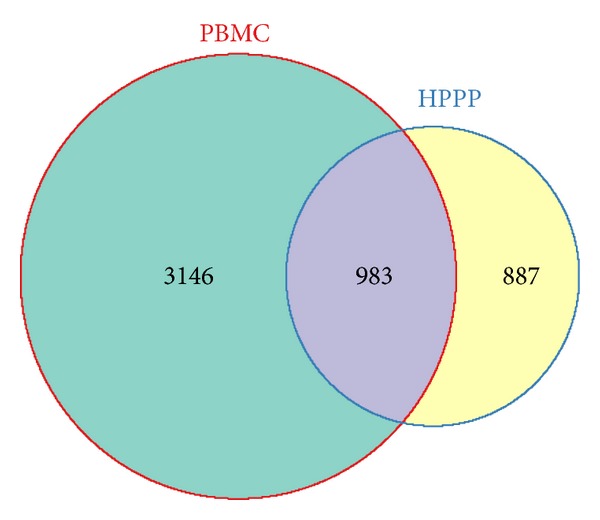
Venn diagram demonstrating the overlap between the PBMC and plasma (HPPP) proteome.

This set of PBMC proteins can be compared to previous data of this cellular part of the human blood on one hand and also on the other hand to the proteome of the body fluid plasma. By performing the latter comparison, we illustrate and describe the additional information provided by (the cellular) PBMC samples for clinical blood proteomics. We selected the human plasma proteome database (HPPP) as the benchmark plasma protein catalogue for comparison [15]. As expected, the total proteomes of the PBMC proteome and the plasma proteome differ significantly. In total 4129 different protein groups were detected in PBMCs compared with 1929 plasma protein groups reported in HPPP. The proteins detected in PBMCs as one cellular compartment of blood deliver more than the double amount of proteins reported for plasma. However, of higher importance than the widely known fact that cellular proteomes in general deliver higher numbers of proteins than plasma is that the two sample types—PBMCs and plasma—encode different proteins. The Venn diagram in Figure 3 shows that 983 protein groups are common between PBMCs and HPPP, whereas 3146 and 887 protein groups are unique for PBMC and HPPP, respectively. More than 3000 proteins are detected only in PBMCs which illustrates that the focus of detected proteins is substantially different and significantly richer as a source of biomarkers.

**Figure 4:**
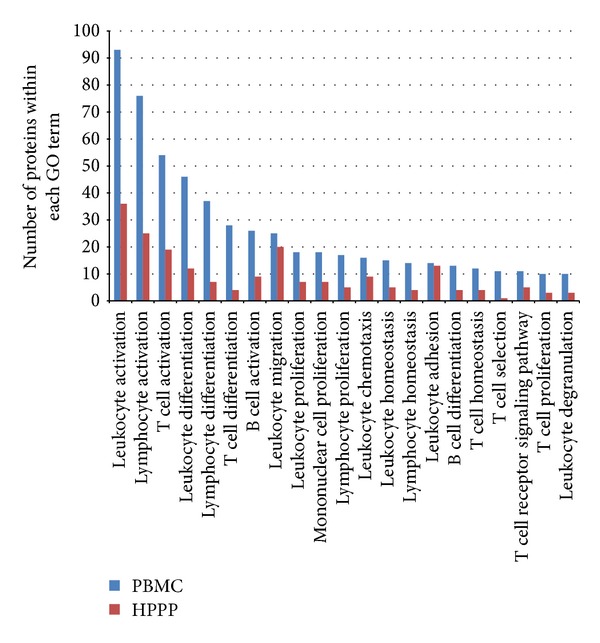
Protein count comparison of PBMC and plasma (HPPP) proteome focusing on PBMC relevant biological processes (GO).

As PBMCs are mainly comprised of monocytes, T cells, B cells, natural killer (NK) cells, and dendritic cells, many of the proteins present in PBMCs play important roles in immune system monitoring relevant events and response in an inflammatory manner. To evaluate how much of this important disease-relevant information is retained in blood plasma, we further evaluated the difference of the PBMC and human plasma proteomes and compared gene ontology biological processes terms. We have selected PBMC relevant terms (like T cell, B cell, lymphocyte, mononuclear, and leukocyte) and analyzed the respective protein counts in the PBMC and plasma (HPPP) proteome.  Figure 4 shows that proteins being relevant to the typical PBMC functions are highly enriched in the PBMC data. This demonstrates that proteins playing important roles in the immune system and in connected diseases can be readily analyzed in PBMCs with our approach, whereas (as expected) plasma contains a lower number of proteins carrying respective immune-relevant information.

Under pathological conditions, membrane proteins represent one of the most interesting classes of proteins among disease biomarker candidates and, because of their exposed surface localisation, represent potential drug targets [22]. We have found 792 proteins in PBMCs and only 245 in plasma (HPPP) containing the gene ontology cellular component term “intrinsic to membrane” highlighting the potential of the PBMC workflow for elucidating the function of membrane proteins of this blood cellular compartment.

**Figure 5:**
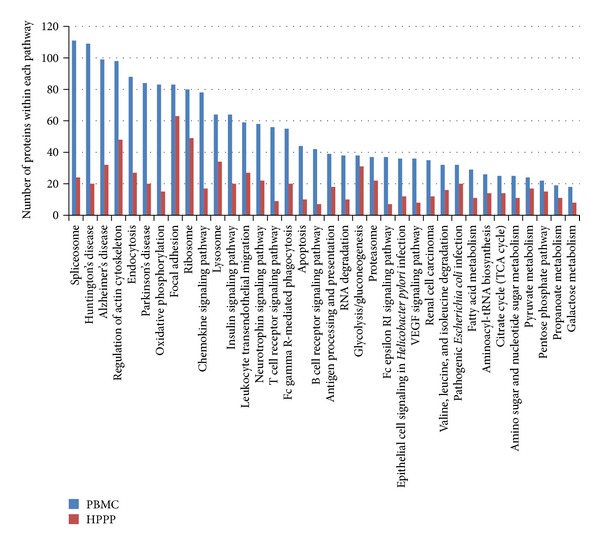
Enriched KEGG pathways for PBMC proteome and in comparison to plasma (HPPP) (*P* < 0.01).

**Figure 6:**
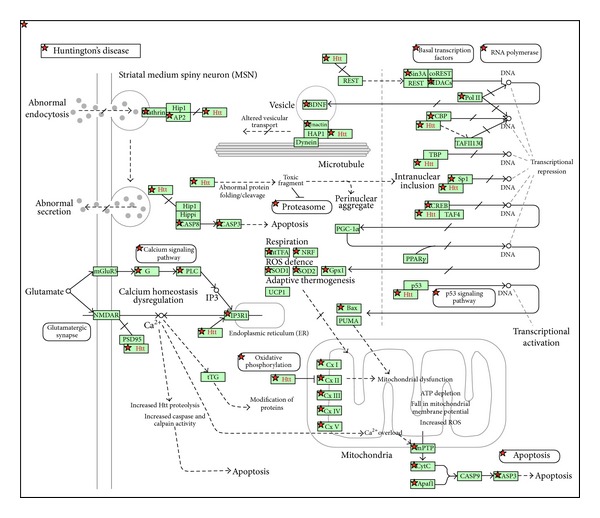
KEGG pathway for Huntington's disease (proteins identified in our study of the PBMC proteome are highlighted by red stars).

To further investigate the potential of PBMCs in clinical proteomics, we have compared the involvement of PBMC proteins in biochemical pathways from the KEGG database. The comparison in Figure 5 summarizes proteins significantly enriched in number in the PBMC sample (compared to the plasma proteome). Striking is the high coverage of neurodegenerative disease pathways like Huntington's disease, Alzheimer's disease, and Parkinson's disease in the PBMC sample. Further, signalling pathways like those connected to chemokines, insulin, neurotrophins, T cell receptor, B cell receptor, and VEGF are covered by far more proteins in PBMCs compared to plasma. The PBMC protein data cover neurological disease specific pathways for Alzheimer's, Parkinson's, and Huntington's disease in an extraordinary manner. This is illustrated for Huntington's disease in Figure 6, where Huntington's disease pathway highlights the excellent proteomics coverage. It is well known that in patients with neurological diseases immunophenotypic changes in PBMC are common; for example, in Alzheimer's disease patients the involvement of systemic immunity has been demonstrated by proteomic changes in patient-derived PBMCs [23, 24]. Also for neurodevelopmental disorders such as autism spectrum disorders (ASD) the usefulness of PBMC analysis was demonstrated by the measurement of lowered amounts of IL-23 [25]. Also, the proteomic analysis of PBMCs from amyotrophic lateral sclerosis (ALS) patients identified combinations of protein biomarkers that can distinguish, with high discriminatory power, patients from healthy controls [26].

Haudek-Prinz et al. have comprehensively characterized the proteome signature of inflammatory-activated isolated T cells, monocytes, and PBMCs [1]. The methodological approaches they used were 2D-PAGE and MS-shotgun analysis and they show that PBMC activation is accompanied by, for example, NAMPT and PAI2. They define a T cell specific inflammation signature, which includes IRF-4, GBP1, and GBP5. The corresponding monocyte activation signature includes the proteins PDCD5, IL1RN, and IL1B. In the light of these marker data, we have analyzed our data set obtained from PBMCs and see that we detect most of the evaluated activation markers described by Haudek-Prinz et al. (over 80% of proteins from Tables 1 and  2 in [1]). Among these proteins are, for example, NAMPT as a marker of PBMC activation, GBP-1 as a marker candidate described for lymphocyte activation, PDCD5 as one marker described for monocyte activation, and many more. Therefore, our method is useful for measuring inflammatory-activation processes in PBMCs.

As reviewed by Shipkova and Wieland, surface proteins are the mainstay analytes used to measure activation or cell proliferation of, for example, lymphocytes [27]. Activated T cells express surface receptors like CD25 (IL-2 receptor) and CD71 (transferrin receptor) or also costimulatory molecules (e.g., CD26, CD27, CD28, CD30, CD154 or CD40L, and CD134). A range of methods exist for both in vitro and ex vivo investigations with either isolated lymphocytes or whole blood to measure activation or proliferation of specific cells. PBMCs are often studied using flow cytometric analysis. Here, cell surface proteins are used for identification and investigation of immunophenotyping of cells [27]. The nomenclature for such proteins is defined by cluster of differentiation names (CD nomenclature). CD molecules act in numerous ways, for example, as receptors or ligands [28]. To assess the value of our PBMC proteome data set concerning these proteins, we have filtered the protein names for CD proteins and found that we detect 36 proteins that have a CD name assigned (Supplemental Table S2). A further protein that is frequently used to measure activation of PBMCs is PCNA [29]—this protein is also detected in our analysis (Supplemental Table S2). These examples serve to illustrate that our data reflect the broad range of proteins being of relevance in PBMCs activation and proliferation processes. Thus, the proteomic approach is excellently suited for the study of PBMCs and the attached physiological roles like activation studies and immunophenotyping. By using a TMT-based quantitative workflow, biomarker discovery projects for quantification of up to 10 samples in parallel are enabled [30]. The method setup is thus suited to significantly extend the range of molecular markers defined for specific activation states of all cell types contained in PBMCs, which then may be exploited for focused activation studies where a complete panel of activation markers will be measurable in a multiplex manner. Methodologically this can also be achieved by transferring newly identified or already established protein markers into a TMT-selected reaction monitoring (SRM) based approach. Similar approaches are often used to confirm protein regulations found during discovery studies [31].

## 4. Conclusions

This study represents a benchmark in deciphering the PBMC proteome as we provide the deepest insight described so far. The identified proteome defines the scope that enables us to characterise the PBMCs in detail. We deduce not only that blood as source sample delivers the biological fluids plasma or serum as important sample type for proteomics, but also that PBMCs as a blood-derived cellular sample will represent a valuable sample for future clinical biomarker studies. We aim to establish a blood-based database of PBMC related proteins in order to facilitate the discovery of protein biomarkers that can be used for early diagnosis and monitoring the progression and response to treatment of various diseases using a minimally invasive sample.

## Supplementary Material

The supplementary material contains 5 sheets: Table “S1-protein groups” contains all details about identified protein groups. Table “S1-peptide groups” contains the details about identified peptide groups. Table “S1-PSMs” contains the details about all Peptide Spectral Matches. The Table “S2-Filtered Proteins” summarizes information on selected proteins of interest. Table “S3-Method_variation_overlap” contains an exemplary calculation of overlap between different LC-MS-acquisition methods.

## Figures and Tables

**Table 1:** Summary of all LC-MS runs depicting the applied fractionation and liquid chromatography, as well as MS method (standard is referring to the description in Section 2).

LC-MS run	Fractionation method	LC method	MS method
2 h LC HCD 1	No	5–30% ACN over 90 minutes	Standard
2 h LC HCD exclusion list	No	5–30% ACN over 90 minutes	Exclusion list
2 h LC HCD 2	No	5–30% ACN over 90 minutes	Standard
2 h LC HCD 3	No	5–30% ACN over 90 minutes	Standard
2 h LC HCD 4	No	5–30% ACN over 90 minutes	Standard
2 h LC HCD 5	No	5–30% ACN over 90 minutes	Standard
2 h LC HCD (*m*/*z* 300–1500) 1	No	5–30% ACN over 90 minutes	*m*/*z* 300–1500
2 h LC HCD (*m*/*z* 300–1500) 2	No	5–30% ACN over 90 minutes	*m*/*z* 300–1501
2 h LC HCD (*m*/*z* 300–1500) 3	No	5–30% ACN over 90 minutes	*m*/*z* 300–1502
2 h LC HCD (*m*/*z* 300–1500) 4	No	5–30% ACN over 90 minutes	*m*/*z* 300–1503
2 h LC HCD (*m*/*z* 300–536)	No	5–30% ACN over 90 minutes	*m*/*z* 300–536
2 h LC HCD (*m*/*z* 531–679)	No	5–30% ACN over 90 minutes	*m*/*z* 531–679
2 h LC HCD (*m*/*z* 674–840)	No	5–30% ACN over 90 minutes	*m*/*z* 674–840
2 h LC HCD (*m*/*z* 835–1500)	No	5–30% ACN over 90 minutes	*m*/*z* 835–1500
4 h LC HCD 1	No	5–30% ACN over 215 minutes	Standard
4 h LC HCD 2	No	5–30% ACN over 215 minutes	Standard
6 h LC HCD 1	No	5–30% ACN over 335 minutes	Standard
6 h LC HCD 2	No	5–30% ACN over 335 minutes	Standard
8 h LC HCD 1	No	5–30% ACN over 455 minutes	Standard
8 h LC HCD 2	No	5–30% ACN over 455 minutes	Standard
SCX fraction 1 2 h LC HCD	SCX chromatography	5–30% ACN over 90 minutes	Standard
SCX fraction 2 2 h LC HCD	SCX chromatography	5–30% ACN over 90 minutes	Standard
SCX fraction 3 2 h LC HCD	SCX chromatography	5–30% ACN over 90 minutes	Standard
SCX fraction 4 2 h LC HCD	SCX chromatography	5–30% ACN over 90 minutes	Standard
SCX fraction 5 2 h LC HCD	SCX chromatography	5–30% ACN over 90 minutes	Standard
SCX fraction 6 2 h LC HCD	SCX chromatography	5–30% ACN over 90 minutes	Standard
SCX fraction 7 2 h LC HCD	SCX chromatography	5–30% ACN over 90 minutes	Standard
SCX fraction 8 2 h LC HCD	SCX chromatography	5–30% ACN over 90 minutes	Standard
SCX fraction 9 2 h LC HCD	SCX chromatography	5–30% ACN over 90 minutes	Standard
SCX fraction 1 2 h LC HCD exclusion list	SCX chromatography	5–30% ACN over 90 minutes	Exclusion list
SCX fraction 2 2 h LC HCD exclusion list	SCX chromatography	5–30% ACN over 90 minutes	Exclusion list
SCX fraction 3 2 h LC HCD exclusion list	SCX chromatography	5–30% ACN over 90 minutes	Exclusion list
SCX fraction 4 2 h LC HCD exclusion list	SCX chromatography	5–30% ACN over 90 minutes	Exclusion list
SCX fraction 5 2 h LC HCD exclusion list	SCX chromatography	5–30% ACN over 90 minutes	Exclusion list
SCX fraction 6 2 h LC HCD exclusion list	SCX chromatography	5–30% ACN over 90 minutes	Exclusion list
SCX fraction 7 2 h LC HCD exclusion list	SCX chromatography	5–30% ACN over 90 minutes	Exclusion list
SCX fraction 8 2 h LC HCD exclusion list	SCX chromatography	5–30% ACN over 90 minutes	Exclusion list
SCX fraction 9 2 h LC HCD exclusion list	SCX chromatography	5–30% ACN over 90 minutes	Exclusion list
SCX fraction 10 2 h LC HCD exclusion list	SCX chromatography	5–30% ACN over 90 minutes	Exclusion list
SCX fraction 1 2 h LC (15–30% ACN)-optimized LC gradient	SCX chromatography	15–30% ACN over 90 minutes	Standard
SCX fraction 2 2 h LC (15–30% ACN)-optimized LC gradient	SCX chromatography	15–30% ACN over 90 minutes	Standard
SCX fraction 3 2 h LC (5–30% ACN)-optimized LC gradient	SCX chromatography	5–30% ACN over 90 minutes	Standard
SCX fraction 4 2 h LC (0–30% ACN)-optimized LC gradient	SCX chromatography	0–30% ACN over 90 minutes	Standard
SCX fraction 5 2 h LC (0–30% ACN)-optimized LC gradient	SCX chromatography	0–30% ACN over 90 minutes	Standard
SCX fraction 6 2 h LC (5–30% ACN)-optimized LC gradient	SCX chromatography	5–30% ACN over 90 minutes	Standard
SCX fraction 7 2 h LC (10–30% ACN)-optimized LC gradient	SCX chromatography	10–30% ACN over 90 minutes	Standard
SCX fraction 8 2 h LC (5–30% ACN)-optimized LC gradient	SCX chromatography	5–30% ACN over 90 minutes	Standard
SCX fraction 9 2 h LC (0–30% ACN)-optimized LC gradient	SCX chromatography	0–30% ACN over 90 minutes	Standard
SCX fraction 10 4 h LC (5–30% ACN)-optimized LC gradient	SCX chromatography	5–30% ACN over 215 minutes	Standard

## Abbreviations

ACN: Acetonitrile
Da: Dalton
DTT: Dithiothreitol
EDTA: Ethylenediaminetetraacetic acid
IAA: 2-Iodoacetamide
GO: Gene ontology
LC: Liquid chromatography
MS: Mass spectrometry
PBMC: Peripheral blood mononuclear cells
pFDR: Positive false discovery rate
ppm: Parts per million
RP: Reversed phase
SCX: Strong cation exchange
SDS: Sodium dodecyl sulfate
SRM: Selected reaction monitoring
TCEP: Tris(2-carboxyethyl)phosphine
TEAB: Triethylammonium bicarbonate
TFA: Trifluoroacetic acid
Tris: Tris(hydroxymethyl)aminomethane
2-DE: Two-dimensional gel electrophoresis
TMTs: Tandem mass tags.
